# Errors as a primary cause of late-life mortality deceleration and plateaus

**DOI:** 10.1371/journal.pbio.2006776

**Published:** 2018-12-20

**Authors:** Saul Justin Newman

**Affiliations:** Research School of Biology, The Australian National University, Acton, ACT, Australia; Charité—Universitätsmedizin Berlin, Germany

## Abstract

Several organisms, including humans, display a deceleration in mortality rates at advanced ages. This mortality deceleration is sufficiently rapid to allow late-life mortality to plateau in old age in several species, causing the apparent cessation of biological ageing. Here, it is shown that late-life mortality deceleration (LLMD) and late-life plateaus are caused by common demographic errors. Age estimation and cohort blending errors introduced at rates below 1 in 10,000 are sufficient to cause LLMD and plateaus. In humans, observed error rates of birth and death registration predict the magnitude of LLMD. Correction for these sources of demographic error using a mixed linear model eliminates LLMD and late-life mortality plateaus (LLMPs) without recourse to biological or evolutionary models. These results suggest models developed to explain LLMD have been fitted to an error distribution, that ageing does not slow or stop during old age in humans, and that there is a finite limit to human longevity.

## Author summary

In diverse species, mortality rates increase with age at a relatively fixed rate within populations. However, recent discoveries have suggested this relationship breaks down in advanced old age, with mortality rate increases slowing and even reaching a plateau. This late-life mortality deceleration has initiated sustained debate on the cause of late-life deceleration and plateaus. Proposed explanations include evolutionary patterns, the exhaustion of selective pressure, population heterogeneity, and even the cessation of the ageing process. Here, I demonstrate that apparent late-life mortality decelerations and plateaus can be generated by low-frequency errors. I then reveal how indicators of demographic data quality predict the magnitude of late-life mortality deceleration and the existence of late-life plateaus in human populations. These findings suggest that human late-life mortality plateaus are largely, if not entirely, artefacts of error processes. As a result, late-life mortality plateaus and decelerations may be explained by error patterns in humans and many other species without invoking complex biological, heterogeneity, or evolutionary models. This finding has immediate consequences for demographic modelling, evolutionary biology, and the projected upper limits of human and nonhuman life.

## Introduction

**Fig 1:**
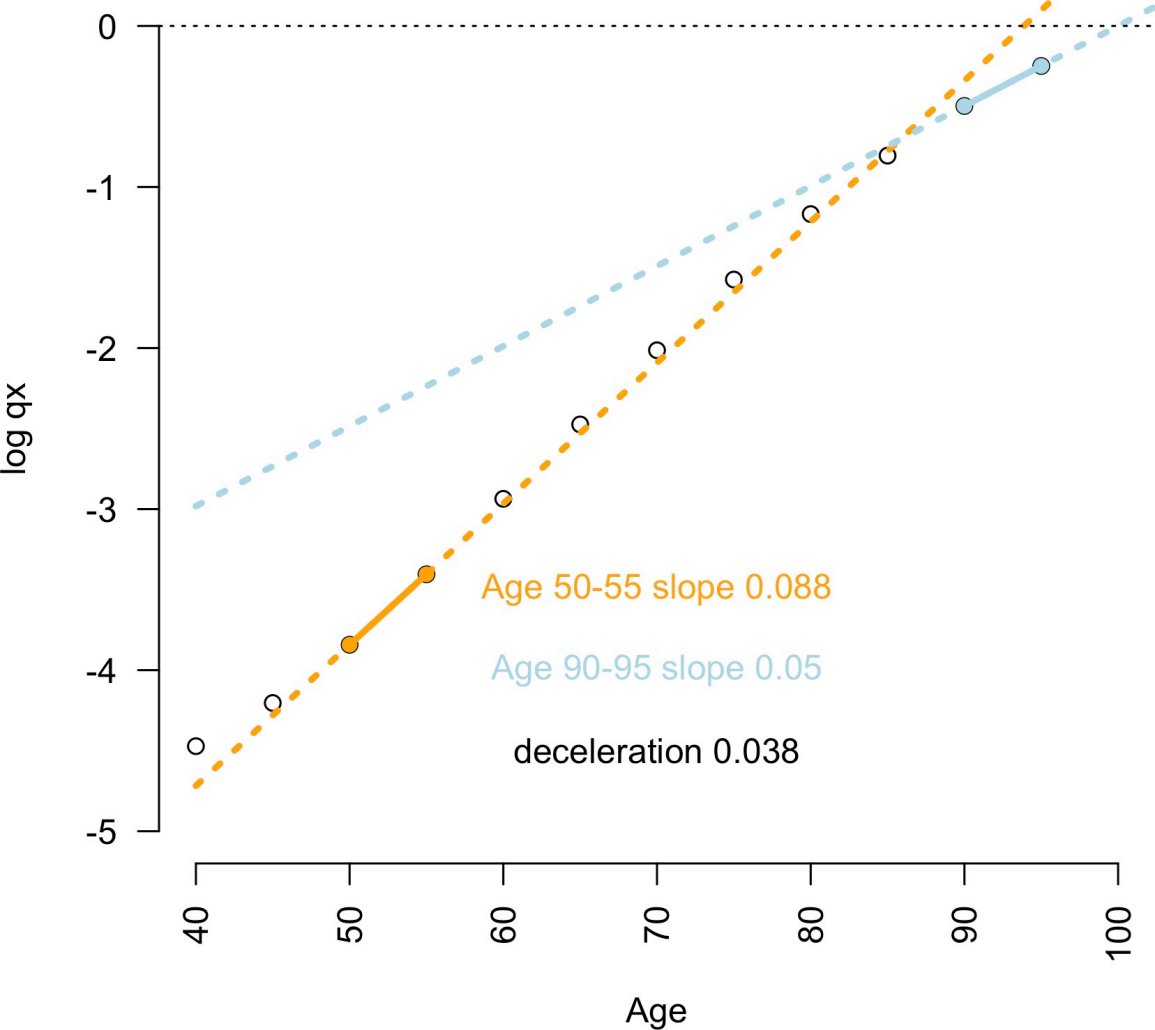
LLMD measured by the gap between mid-life and late-life mortality rate increase. Pooled global data on the age-specific probability of death *q*_*x*_ (2015 data; female population) show the relatively slower rate of late-life mortality acceleration (ages 90–95; blue line) compared with mid-life mortality (ages 50–55; orange line) in humans. The difference between these slopes indicates the magnitude of LLMD. Underlying data can be found in S1 Data. LLMD, late-life mortality deceleration.

The age-specific probability of death follows diverse, often species-specific curves. In several species, including humans, rates of mortality increase with age have been observed ‘flattening’ in advanced old age [1,2]. In some cases, this late-life mortality deceleration (LLMD) is sufficient to cause a ‘levelling off’ or plateau in the probability of death at advanced ages (Fig 1). LLMD and late-life mortality plateaus (LLMPs) have been proposed to cause the respective slowing or cessation of biological ageing at advanced ages [2] and, respectively, increase and remove the upper limits of survival in humans [3,4].

These findings have led to continuing debate on the biological meaning, magnitude, and importance of LLMDs and LLMPs. Several hypotheses and models have been proposed to explain the observation of LLMPs and LLMDs in diverse taxa, such as population heterogeneity, density effects, and evolutionary theories [5–9]. In parallel, these observations have led to the development and widespread use [10] of demographic models, such as the Kannisto old-age–mortality model [11,12], that assume a priori the existence of LLMPs.

However, there is evidence that LLMPs can result from diverse statistical errors, such as the pooling of human cohorts, choice of mortality rate metric or time interval [13], and missing death certification or age-reporting errors [12,14,15]. Furthermore, in any species with finite upper limits of life, both random and nonrandom error distributions will necessarily favour the inclusion of younger individuals amongst the oldest survivable age categories, reducing the subsequent probability of death calculated for these ages. As a result, deformation of late-life mortality by biodemographic errors may provide a general explanation of LLMDs and LLMPs.

Therefore, understanding late-life mortality patterns requires consideration of the effect of age-coding errors and whether the late-life patterns of mortality rates in humans may represent combined outcomes of measurement and sampling errors.

Here, it is revealed how diverse demographic errors deform the age-specific mortality curve and the hazard rate (S1 Code), causing LLMDs and LLMPs in the absence of other effects. In humans, the error rate of demographic sampling, completeness of birth and death records, and development and income indicators all predict the magnitude of LLMD. Correcting for these factors eliminates LLMDs and LLMPs, suggesting these patterns are caused by sampling and measurement error and not by biological or evolutionary factors.

## Results

While generally insufficient to cause LLMPs, with 27 populations (9.6%) having a late-life slope of mortality below 0.1, some degree of LLMD was common across human populations (S1 Code). Previous discussions have presented this late-life deceleration and any resultant LLMPs as a biologically significant evolved pattern in humans [5,9]. However, we found evidence to suggest that LLMDs and LLMPs are a result of recording errors and sampling biases.

**Fig 2:**
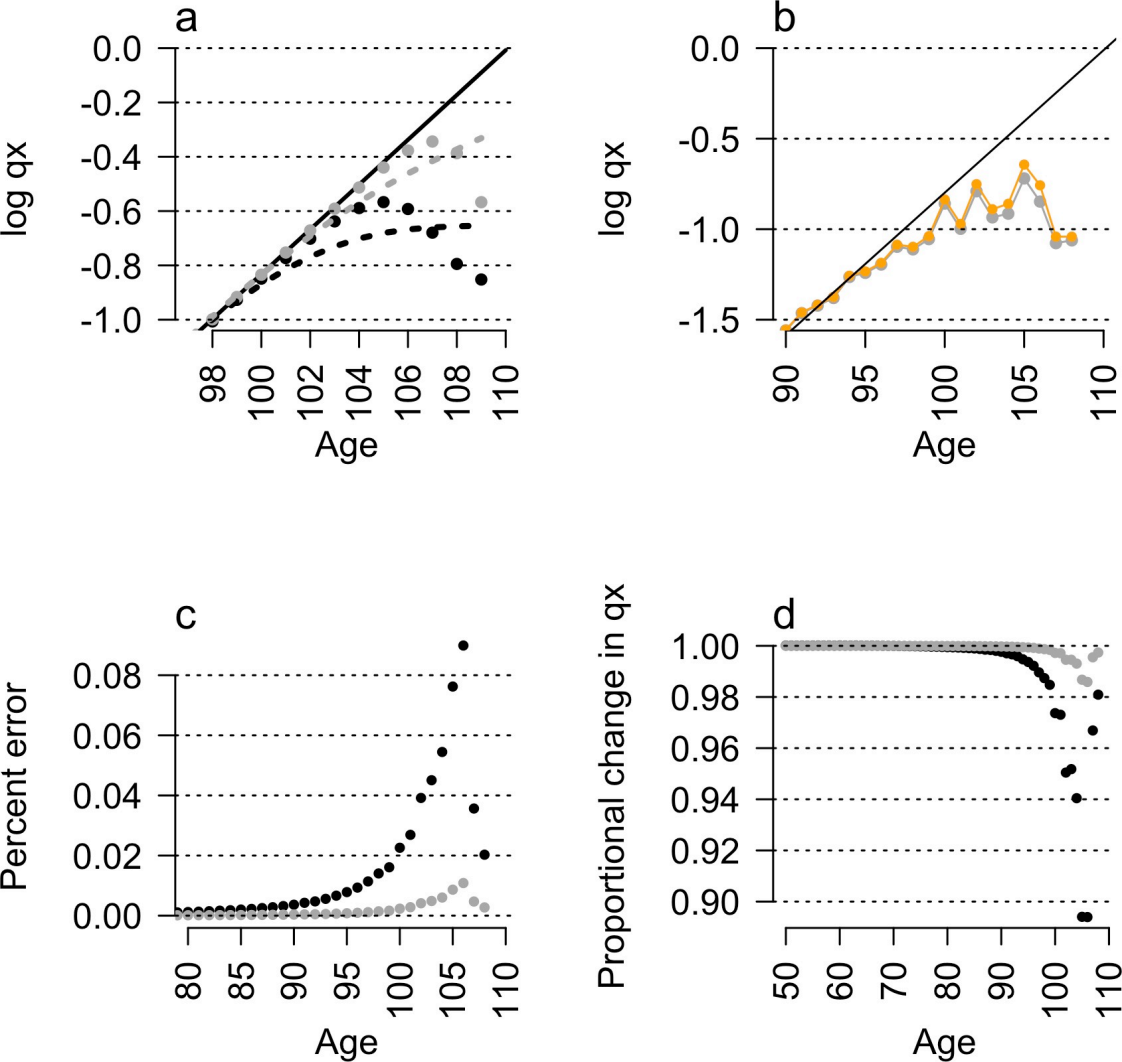
Random errors cause LLMDs and LLMPs. (a) Introducing random age-reporting errors into a log-linear model of mortality (solid black line) artificially lowers the age-specific probability of death *q*_*x*_ (points) in late life, causing LLMD and LLMPs (dotted lines). (b) These simulated effects often reflect late-life mortality patterns observed in real data, for example, shown here in Jeanne Calment’s birth cohort (orange). Introducing age-coding errors by randomly reassigning individuals between observed cohorts (b) further increases rates of mortality deceleration, (c) increases proportionally larger errors in the calculated probability of death, and (d) greatly reduces the probability of death at advanced ages. Exact effect of errors calculated at a probability *p* = 0.001 (grey) and *p* = 0.0001 (black), data in (a) fitted by locally smoothed splines (dashed lines). Underlying data can be found in S2 Data. LLMD, late-life mortality deceleration; LLMP, late-life mortality plateau.

Random error in reported age generates LLMPs and LLMDs in the absence of other effects (Fig 2A–2D; S1 Code; S1 Text). Introducing random 10-year age coding or cohort-blending errors into a null, log-linear mortality model (Fig 2A) or into real cohorts (Fig 2B–2D) generated LLMDs and, when aggregated into quinquennial data or fitted by smoothed splines, mortality plateaus (Fig 2A; S1 Code; S1 Text).

Random error in the allocation of individuals to cohorts and in certified ages at death causes errors to constitute a substantial and increasing proportion of deaths in old-age cohorts, even for low (*p* < 10^−5^) rates of error (Fig 2D). Higher survival rates in younger individuals, included by error into older age groups, enrich representation of age overestimation errors as a cohort ages. Conversely, individuals with under-reported ages suffer higher mortality and constitute a falling proportion of errors with age.

For example, the average effect of a 1 in 1,000 age-coding error rate can be introduced into Jeanne Calment’s birth cohort at age 50. By age 100, virtually no individuals with age underestimation errors remain alive, but the initially rare (0.1%) age overestimation errors constitute over 13% of recorded individuals (Fig 2B–2D; S1 Code). As a result of the increasing frequency of these errors with age, in which individuals are reported as age 100 but have the actual mortality rate of a 90-year-old, mortality rates artificially flatten or decelerate with age. These error patterns result in artefactual LLMDs at less than one-tenth the error rates simulated in previous studies [16] and reveal finer-scale patterns masked by the binning of mortality data into quinquennial and open-ended 100+ age categories [16].

**Fig 3:**
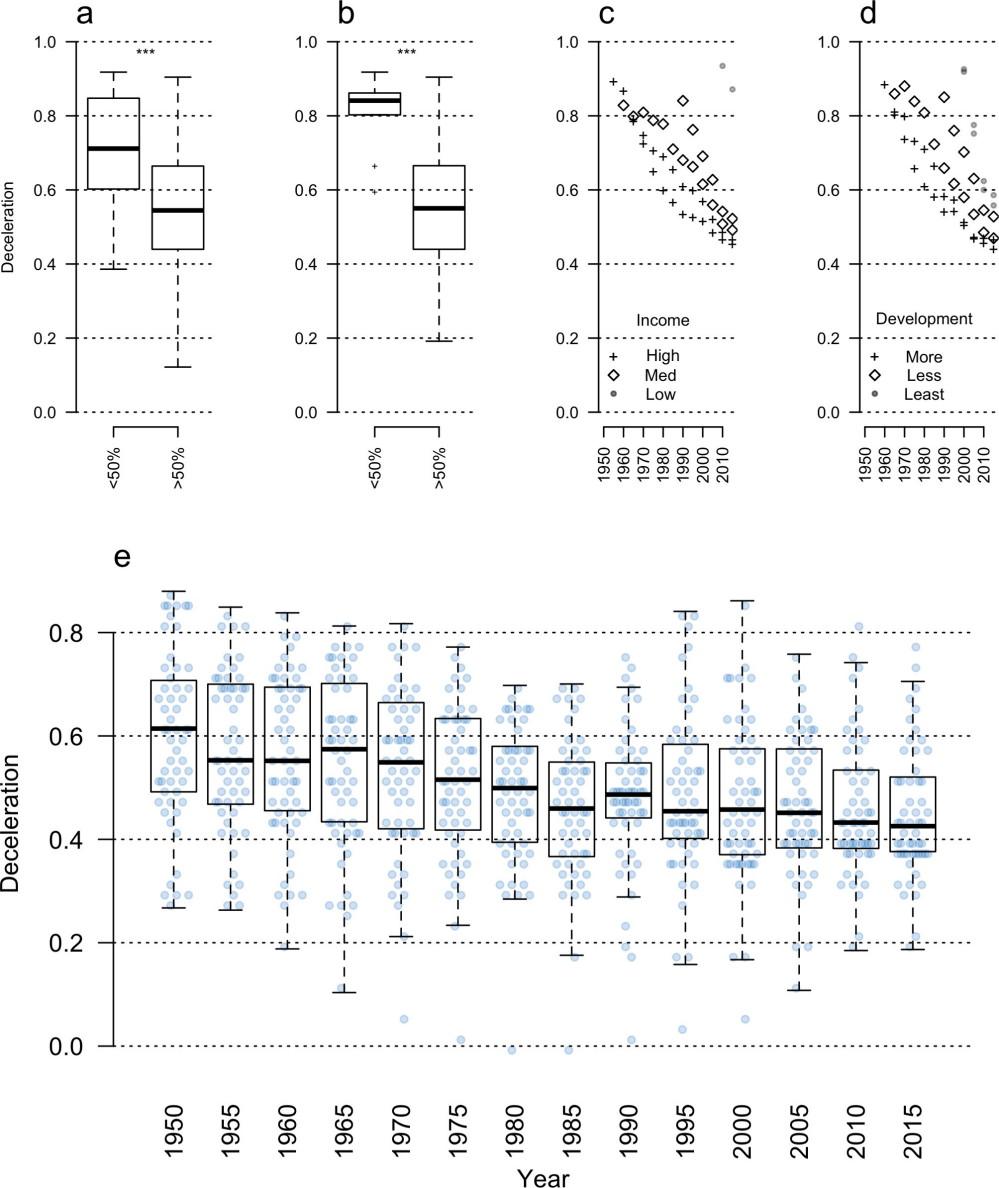
Reduced LLMDs in populations with better population data and higher vital statistics coverage. The rate of LLMD (y-axis) is linked to differences in (a) the fraction of the population with death certificates, (b) the fraction of the population with birth certificates, (c) per capita gross domestic product, and (d) levels of population development (Bonferroni-corrected pairwise *t* test; asterisks indicate *p* < 0.001). In populations with continuous records of late-life mortality (e), mortality deceleration rates have fallen by 25% since 1950, alongside gains in civil registration rates (N = 55). Underlying data can be found in S3 Data. LLMD, late-life mortality deceleration.

The magnitude of LLMDs and the frequency of LLMPs are predicted by broad indicators of vital statistics data quality and by demographic error rates in modern populations. LLMPs are weaker in populations with the most complete documentation of vital statistics (Fig 3A and 3B). The richest, most developed, and best-documented countries have the smallest LLMDs (Fig 3C and 3D; S1 Code) and almost never exhibit LLMPs. Furthermore, the degree of LLMD has fallen as coverage for vital statistics has improved over time (Fig 3C–3E). For example, in countries with continuous records of late-life mortality patterns, the magnitude of LLMD has fallen by 25% since 1950 (Fig 3E).

**Fig 4:**
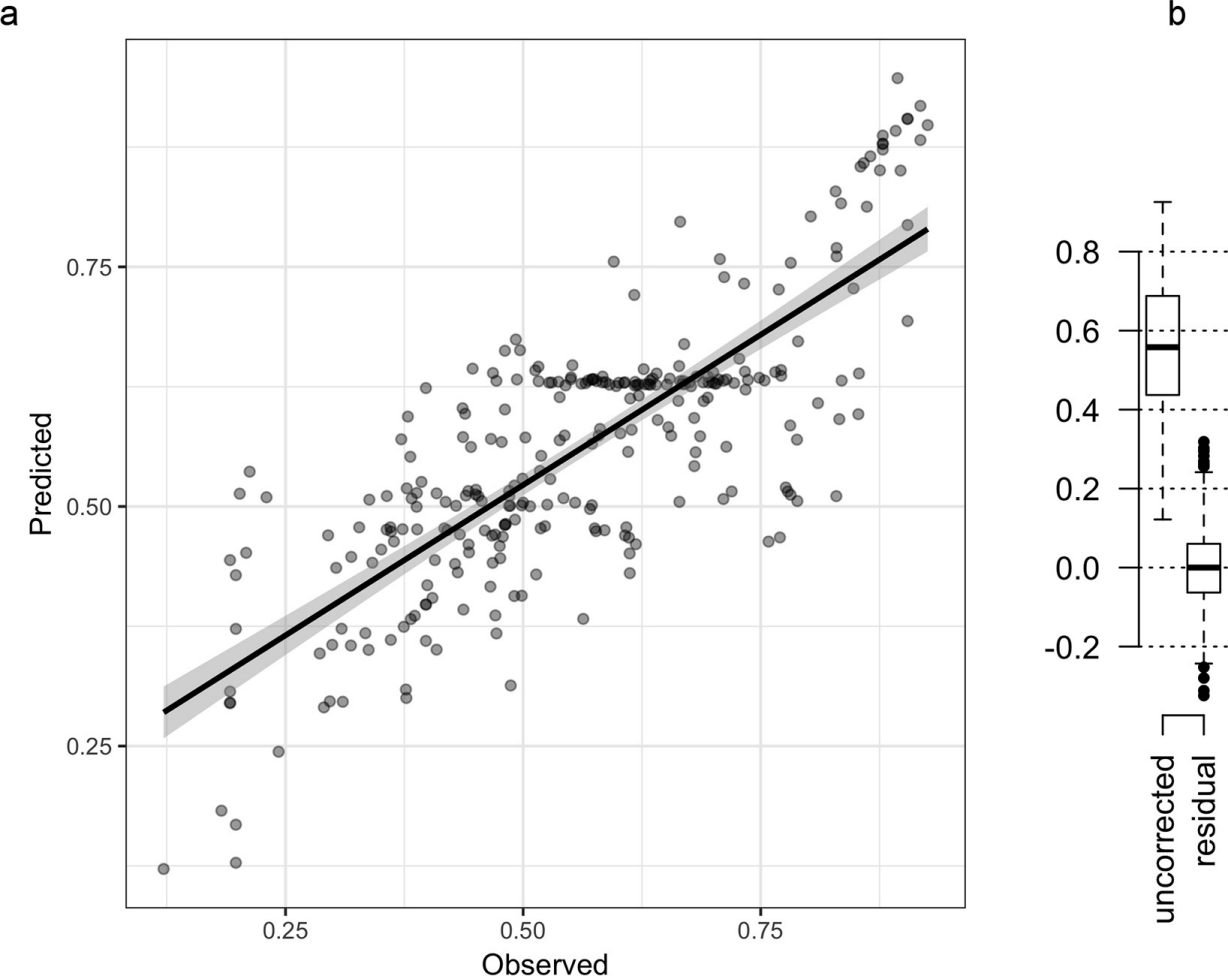
LLMD predicted by a mixed linear model. A mixed linear mixed model (a) constructed using predictors of sampling error rates and continent of sampling explains the majority of human variation in LLMD (Pearson’s r = 0.82; adjusted R^2^ = 0.57; *p* < 10^−6^; N = 280 populations). (b) Correction for these factors eliminates LLMDs in humans. Underlying data can be found in S4 Data. LLMD, late-life mortality deceleration.

While increasing demographic data quality is associated with reduced LLMD across diverse indicators, very few such indicators are required to predict late-life mortality patterns. A mixed linear model fit to the interactive effects of rates of birth and death registration and the continent of residence predicted 60% of the variation in LLMDs (r = 0.6; corrected R^2^ = 0.4; *p* < 0.001; S1 Code). The addition of infant mortality rates to this model, as an indicator of the quality of civil healthcare systems, further improved this model (Fig 4A; r = 0.8; corrected R^2^ = 0.6; *p* < 0.0001; S1 Code). Correcting for just these four variables eliminated LLMDs and LLMPs in human populations (Fig 4B).

The possibility was considered that late life was not strictly defined by a chronological 90-year barrier but by the relative timing of human life history events. In this case, increasing life expectancy may lead to progressively later LLMDs and LLMPs, reducing the LLMD rate observed at age 90 by delaying mortality deceleration to older ages in long-lived populations. In turn, this may lead to the observed relationship between population development and mortality deceleration at ages 90–95.

However, further testing did not support this hypothesis. Both adult (life expectancy at age 40 [e40]) and overall (life expectancy at age 0 [e0]) life expectancy accounted for minimal variation in LLMD rates (r = 0.07 and r = 0.04, respectively). After correcting for variation in adult and overall life expectancy, residual LLMDs followed similar patterns to those observed for uncorrected values (S1 Fig). The strong relationship between error rates and LLMDs in the World Population Prospects (WPP) database [17] was independent of changes in lifespan.

Finally, reanalysis of these data using hazard rates incurred no substantial changes in our findings (S1 Text; S2 Code). The primary change was the loss of recent historical reductions in the scale of LLMDs across the UN data, a trend that reappeared once dramatic changes in lifespan across this period were normalized (S1 Text). The most important finding was retained: generalized linear models fitted to hazard-rate data, using identical predictors to the age-specific mortality-rate model above, eliminated LLMPs and predicted 71% of the variance in the LLMD of human populations (N = 211; *p* < 10^−6^; S1 Text; S2 Code).

## Discussion

Gavrilova and Gavrilov previously provided evidence that late-life plateaus and deceleration are caused by specific demographic measurement errors in developed human populations [12]. Our findings more broadly support this explanation. Error in the recorded age of individuals can cause asymmetric changes in the age-specific probability of death independent of the mechanism of error generation. Previous study of age-coding errors has generated LLMDs through both asymmetric and symmetric error processes [16] yet masked other late-life patterns by binning ages into quinquennial and open-ended age categories. Our data capture these finer-scale patterns and support the position that LLMDs and LLMPs may result from diverse errors such as age-coding errors, mortality under-reporting, or cohort blending [12,14,15].

The capacity of error models to account for observed late-life mortality patterns suggests that models assuming the existence of LLMPs a priori [11] may be poorly informative of late-life biology. Such models may provide a better fit of observed data containing LLMPs and LLMDs caused by errors. However, such ‘best-fit’ models may not accurately inform real patterns of ageing: rather, they may constitute a model overfitted to an error distribution. Therefore, use of these models to rule out upper lifespan limits [3], predict patterns of late-life mortality [10], inform the biology of ageing, and guide global policy on old-age populations may be inappropriate. Indeed, these results support the sentiment that if LLMPs ‘turned out to be generally illusory, much of the demographic modeling of the past two decades would have to be rethought’ [4].

Adjusting for just four indicators of data quality eliminates LLMDs and LLMPs without reference to evolutionary models or population heterogeneity (Fig 4A and 4B). Fitting a linear model of interactive effects between infant mortality, continent of origin, and the fraction of the population with birth and death certificates predicts 80% of the variance in LLMD (Fig 4A). Residual late-life mortality rates are symmetrically distributed around the rate of mid-life mortality acceleration after correcting for these indicators (Fig 4B), suggesting that biological ageing proceeds at a constant rate from mid-life onwards and that any residual variation in late life constitutes sampling noise.

In contrast, biological and evolutionary hypotheses to explain mortality deceleration and plateaus [5,6] are more difficult to reconcile with the observed diversity of late-life mortality patterns. Trait heterogeneity can produce similar late-life patterns to age-coding errors and has been widely considered a possible explanation for LLMPs [8]. However, if LLMD in humans were a product of either selective processes [5,18] or trait heterogeneity [8], it is unclear why population diversity in this trait would change rapidly over short timescales (Fig 3E), correspond to the quality of civil registration (Fig 3A and 3B), and be predicted by development indicators (Fig 3C and 3D).

Furthermore, if late-life mortality patterns reflect underlying biological processes, it is unclear why improvements in civil registration and sample sizes would diminish rather than enhance evidence for LLMD and LLMPs. If late-life mortality patterns were biologically directed, resulting from either adaptive or nonadaptive processes, it should be expected that more accurate and complete records would reinforce evidence for LLMDs and LLMPs. Rather, improvements in the accuracy and completeness of vital statistics lead to the rapid reduction or elimination of these phenomena.

The hypothesis that LLMDs and LLMPs are an artefact of collective sampling and measurement errors provides a simple explanation of late-life mortality patterns and leads to testable predictions. For example, approximately 250,000 youths inflated their ages to enter the 1894–1902 birth cohorts and fight for the United Kingdom in World War I [19]. It can be predicted that such stochastic increases in age inflation may be associated with the concurrent growth of ‘supercentenarian’ cohort sizes and stronger LLMDs. More generally, it can be predicted the magnitude of LLMD within populations will continue to fall over time in proportion to longitudinal improvements in demographic sampling and measurement. In addition, rapid nonlinear improvements in vital registration systems, such as the Danish introduction of double-entry birth registry from 1814–1817 or the introduction of birth certificates to Hawaii and Alaska c.1959 [20], should be associated with corresponding nonlinear reductions in the rate of LLMD within historical data.

Explanations of LLMDs and LLMPs are not necessarily mutually exclusive with evolutionary- and heterogeneity-based explanations. In some populations, room may remain for trait heterogeneity and either adaptive or nonadaptive evolutionary processes to explain residual LLMD (Fig 4B). However, residual LLMDs may also be explained by remnant uncorrected errors in civil registration without reference to complex biological or evolutionary models.

Estimates of late-life mortality necessarily depend on population-wide records generated more than 90 years ago. However, more than a third of global births remained unregistered in 2006 [21]. India and Pakistan still record less than half of all deaths [22], and the half-billion people in Vietnam, Nigeria, Ethiopia, and the Democratic Republic of the Congo have insufficient data to estimate registration rates [22]. Among developed countries, no populations large enough to capture late-life patterns have achieved a 100% registration rate of births and deaths, let alone maintained error-free registration rates for the requisite 90-year period.

Error distributions may also inform similar late-life patterns observed in other species. For example, methods such as mark–recapture and hand counting used to construct wild and laboratory data are susceptible to measurement and sampling error well above the 10^−4^ to 10^−5^ rates sufficient to generate LLMPs. As a result, it can be predicted the increasing capture of biodemographic data will be associated with more frequent observations of LLMPs and LLMDs caused by error.

This prediction is complicated by potential for the evolution of actual LLMD in other species. Evidence in humans does not necessarily inform the late-life mortality of other species beyond highlighting the general capacity for errors to asymmetrically distort late-life mortality. For example, LLMDs were initially detected in manually counted medfly and *Drosophila* cohorts [23,24]. In these initial experiments, mortality deceleration occurred over a small proportion of the total lifespan, a pattern that might have been explained by low-frequency manual counting errors. However, subsequent research has generated ‘late-life’ mortality plateaus that span the majority of the adult female lifespan in *Drosophila*, a pattern unlikely to be generated by cohort or age-coding error distributions [25]. These latter mortality patterns are well supported by evidence and should not be dismissed as error.

Therefore, caution is required to interpret present and future claims of LLMD. Observation of LLMDs and LLMPs may reflect the inherent sensitivity of demographic data to error rather than a shared biological or evolutionary pattern. Alternately, these patterns may represent real changes in age-specific mortality potentially driven by adaptive or nonadaptive evolutionary history, population heterogeneity, behavioural diversity, or the cessation of ageing. Discriminating between such cases will constitute an ongoing challenge in biodemography. While ageing may not ‘stop’ in humans [2], evidence for other taxa requires careful evaluation given the remarkable evolved diversity of age-specific mortality profiles [26].

## Materials and methods

Life table data were generated from a null log-linear increase in the probability of death with age and symmetric decadal age errors seeded into cohorts alive at age 50 (*l*_*50*_) with a probability of 10^−4^, 10^−5^, and 10^−6^ (Fig 2; S1 Code). Life table data were then recalculated using the ‘fmsb’ package [27] in R [28] to include the exact effect of age-coding errors. The resulting change in the late-life mortality was then fitted using both the Kannisto old-age mortality model [11] and a locally weighted smoothed spline (Fig 2A; S1 Code).

Life table data were downloaded from the 2015 edition of the WPP database [17] in R [28]. Pooled quinquennial and annual life table data were obtained for male and female population cohorts from 46 historical populations and subpopulations from the Human Mortality Database [29] (HMD). When necessary, annual life tables were recalculated as quinquennial life tables using the life table conversion functions in the ‘fmsb’ package [27] in R. The slope of log mortality rate increase between ages 90–95 was measured against the slope of log mortality rate increases between ages 50–55, directly capturing the rate of LLMD (Fig 1).

To illustrate the effect of cohort blending errors, data were obtained for the Jeanne Calment annual birth cohort in the HMD [30] and for the 1885 female French birth cohort 10 years later (Fig 2B and 2C). Individuals from the 1885 and 1865 cohorts were blended into the 1875-cohort survivorship curve at ratios of 1 in 1,000 and 1 in 10,000, and the resulting distribution of age-specific mortality rate was recalculated (S1 Code).

To calculate the interaction of error patterns with late-life mortality patterns, WPP data were linked to population size, development, and income groupings included in the WPP 2017 data [17] and obtained from population vital statistics coverage data in the UN Demographic Yearbook [22]. A linear mixed model was used to predict observed LLMD using estimates of population coverage for birth and death rates binned into four pooled ‘quality’ estimates (nonexistent, low [0–50%], medium [50–90%], and high [>90%] vital statistics coverage), infant mortality rates, and the geographic sampling region corresponding to Africa, North and South America, Asia, Europe, and Oceania (S1 Code). When a range of estimates for population birth and death certificate coverage was available, the highest-coverage estimate was used.

These four variables—continent, birth coverage, death coverage, and infant mortality rates—were fitted as interactive effects in a linear mixed model to predict the observed LLMP within a population as an outcome variable (S1 Code). This model was used to correct for the interactive effect of these indicators of demographic sampling quality on mortality rate deceleration (Fig 4B). Furthermore, this model was used to predict the slope of late-life mortality and the size of LLMDs under ideal, but not error-free, conditions for each continent by predicting LLMD and late-life mortality under high vital statistics coverage and zero infant mortality rates (S1 Code).

A linear mixed model was fitted between the rate of LLMD and both the average (e0) and adult (e40) lifespan, and each model used to correct for the effect of increasing population survival with time, income, and development levels. To match the range of ages used in calculating mid-life mortality rate acceleration, adult lifespan was calculated from age 40 onwards to avoid the effect of the ‘accident hump’ in extrinsic mortality rates before this age (S1 Code).

Finally, it has been considered whether LLMPs and LLMDs may be the result of mathematical distortion of the probability of death [31] as life tables approached the natural limit of survival *q* = 1. This possibility was considered somewhat less likely given our use of raw data and exclusion of open-ended terminal age categories. However, to fully ensure this was not the case, a complete reanalysis was conducted using log-transformed hazard-rate data, which are not susceptible to such distortion (S1 Text; S2 Code), and otherwise identical models.

## Supporting information

S1 Code Reproducible R code for the simulation and analysis of late-life patterns in age-specific probability of death curves. (R)

S2 Code Reproducible R code for the simulation and analysis of late-life patterns in age-specific hazard rates, matching the analysis protocols in S1 code as closely as possible. (TXT)

S1 Fig The effect of correcting for average and adult life expectancy variation on LLMDs. Correcting for the weak interaction of average life expectancy (A) or adult life expectancy (B) with LLMD has limited effect on the capacity of data quality to predict mortality rate decelerations. Uncorrected data is shown in Fig 3E. Underlying data can be found in S5 Data. LLMD, late-life mortality deceleration
(TIFF)

S1 Text Complete figure-for-figure reproduction of the manuscript figures using age-specific hazard rates, with annotation, produced by the S2 Code analysis. (PDF)

S1 Data Data underlying Fig 1. (XLSX)

S2 Data Data underlying Fig 2. (XLSX)

S3 Data Data underlying Fig 3. (XLSX)

S4 Data Data underlying Fig 4. (XLSX)

S5 Data Data underlying S1 Fig. (XLSX)

## Abbreviations

e0: life expectancy at age zero
e40: life expectancy at age 40
HMD: Human Mortality Database
LLMD: late-life mortality deceleration
LLMP: late-life mortality plateau
WPP: World Population Prospects
