# Supplementary figures and images for: Errors as a primary cause of late-life mortality deceleration and plateaus
Source: PLoS Biol. 2018 Dec 20;16(12):e2006776. doi: 10.1371/journal.pbio.2006776 (PMC6301557; doi:10.1371/journal.pbio.2006776)

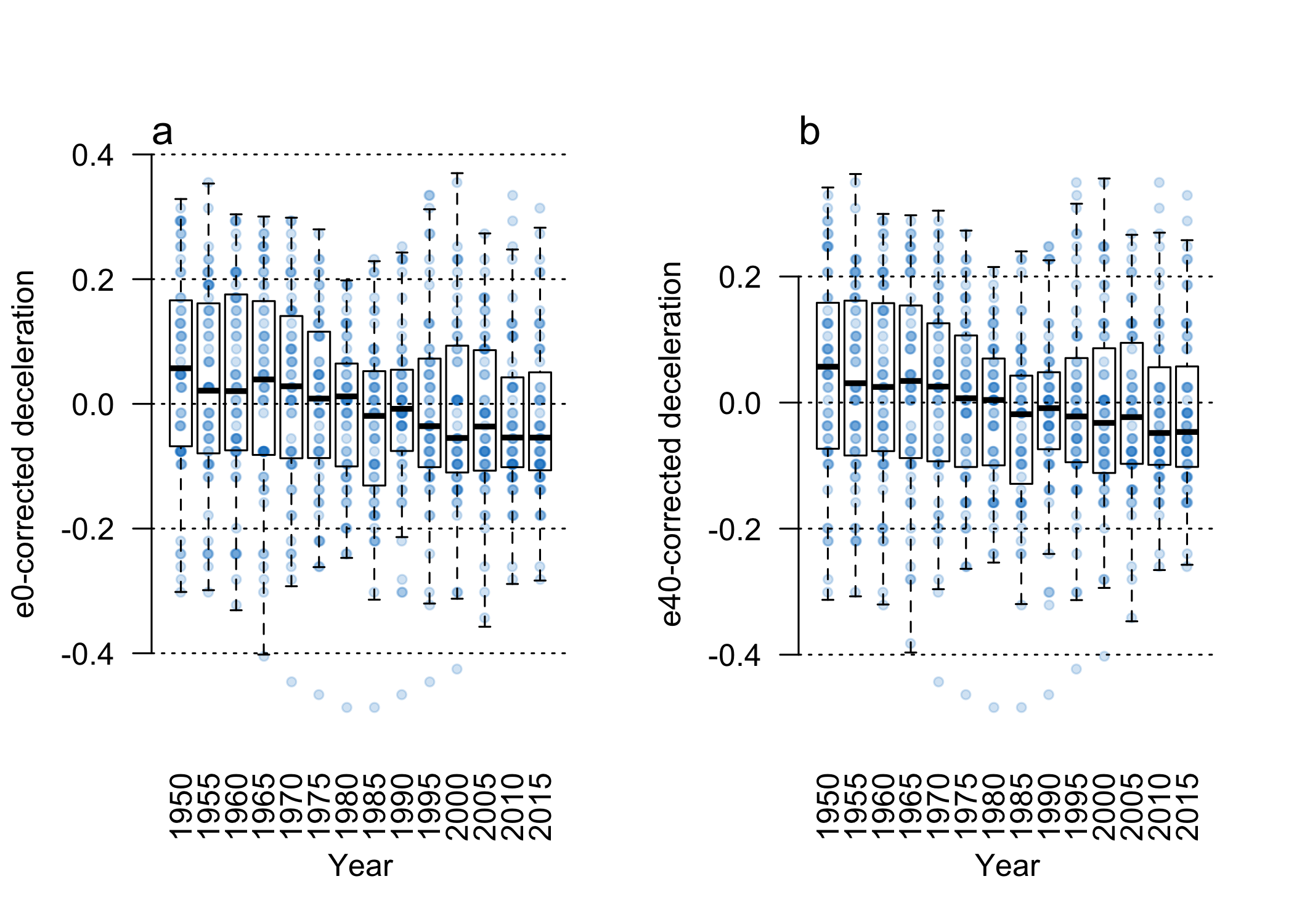

Supplement: S1 Fig — Correcting for the weak interaction of average life expectancy (A) or adult life expectancy (B) with LLMD has limited effect on the capacity of data quality to predict mortality rate decelerations. Uncorrected data is shown in Fig 3E. Underlying data can be found in S5 Data. LLMD, late-life mortality deceleration (TIFF) [file pbio.2006776.s003.tiff]
