# Supplementary material for: Errors as a primary cause of late-life mortality deceleration and plateaus
Source: PLoS Biol. 2018 Dec 20;16(12):e2006776. doi: 10.1371/journal.pbio.2006776 (PMC6301557; doi:10.1371/journal.pbio.2006776)
Supplement: S1 Text — (PDF) [file pbio.2006776.s004.pdf]

Supplementary Information

Reanalysis using hazard rates

This analysis reconstructs, figure-for-figure, the findings in the main manuscript. Differences are noted, especially for Fig. 3e. Methodological differences are included in the annotated code in SI code 2.

The primary difference between use of log age-specific mortality and log hazard rate data is the use of annualized hazard rates, with a corresponding increase in noise, and the trends (or lack thereof) observed over time in the size of hazard rate mortality plateaus.

Collectively, these data indicate the capacity of errors to predict the scale of late-life mortality plateaus and late-life mortality decelerations, measured using either hazard rate data (R^2 = 0.7) or age-specific mortality data (R^2 = 0.8).

While there is an obvious difference in the scale of recent historical trends in these data for figure 3E, there is a considerable degree of deformation in mortality rates across this period. Adjusting for life expectancy or adult life expectancy of a population reveals historical reductions in lifespan-independent late-life mortality patterns.

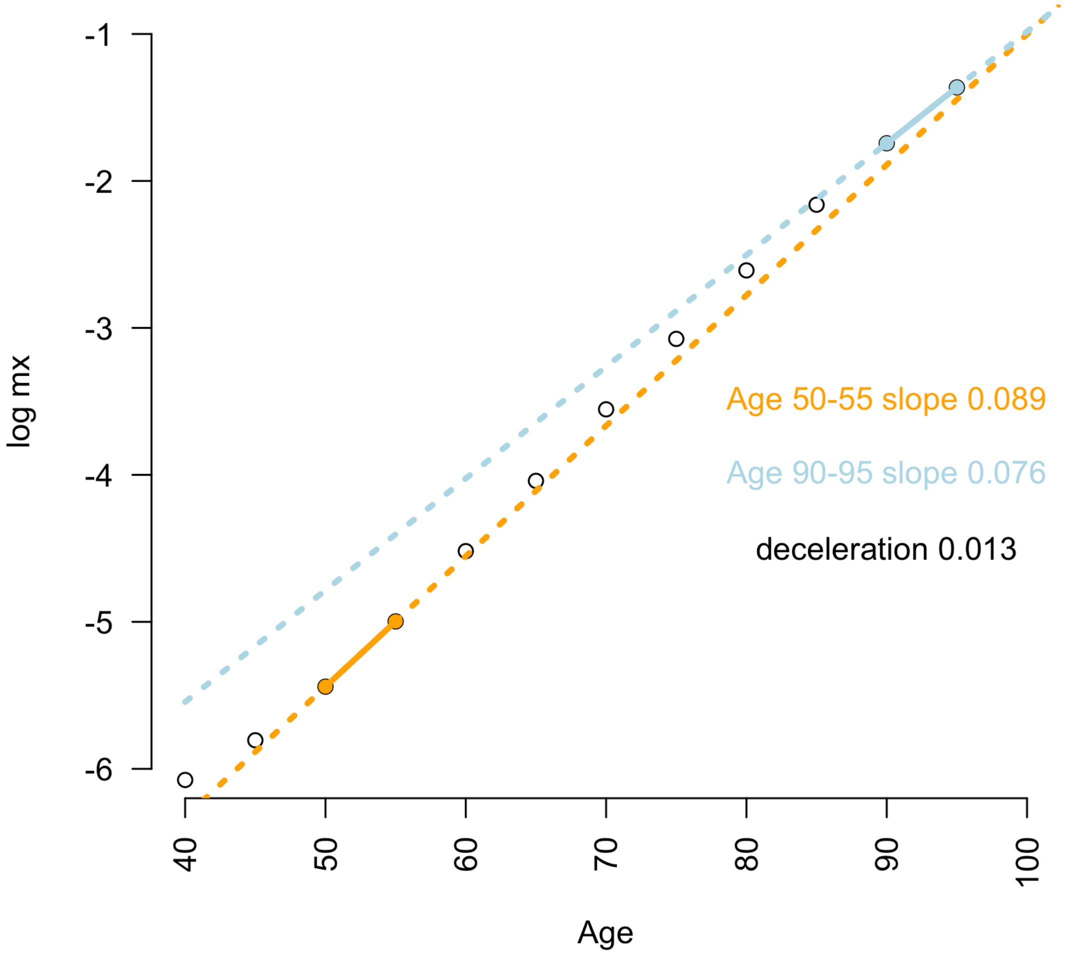

Late-life mortality deceleration measured by the gap between mid-life and late-life mortality rate increase. Pooled global data on the log-transformed hazard rate m_x (2015 data; female population) show the relatively slower rate of late-life mortality acceleration (ages 90-95; blue line) compared with mid-life mortality (ages 50-55; orange line) in humans. The difference between these slopes indicates the magnitude of late-life mortality deceleration.

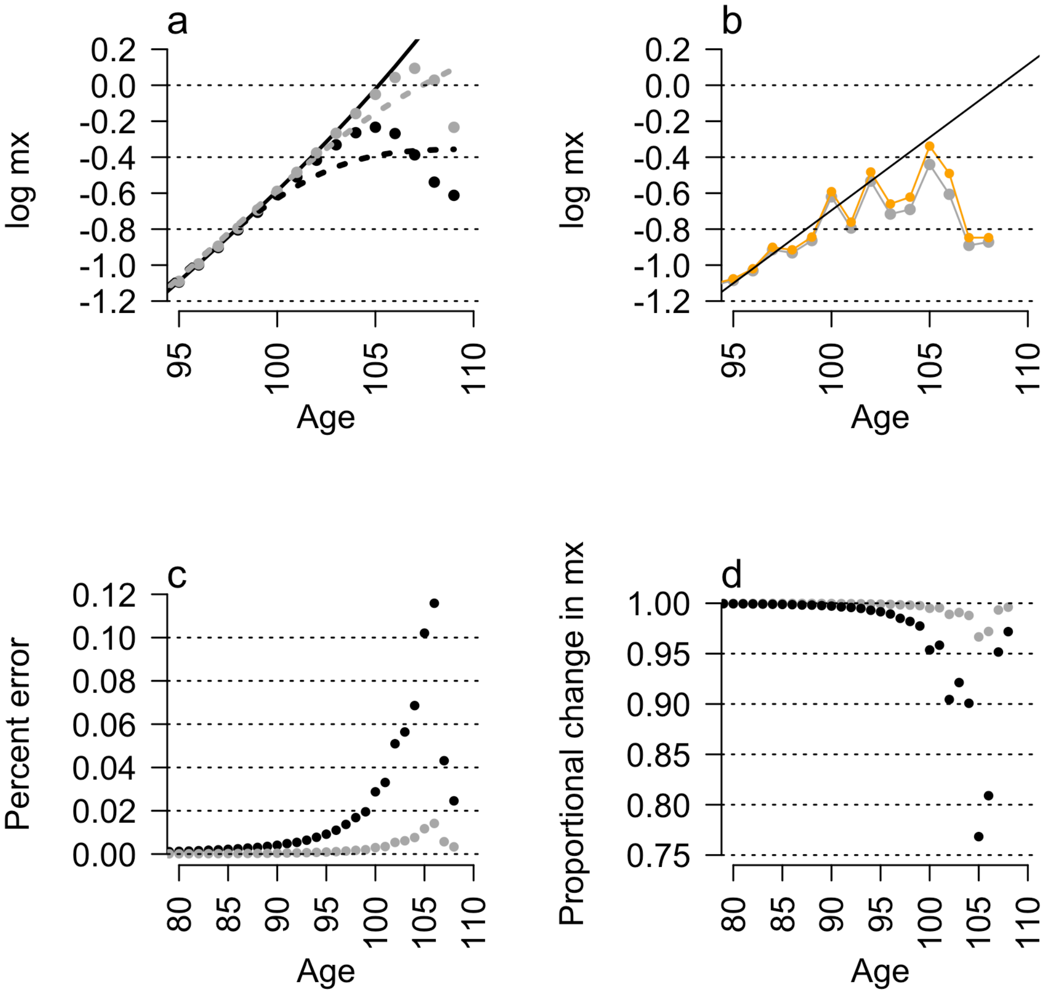

Random errors cause late-life mortality decelerations and plateaus. (A) Introducing random age-reporting errors into a log-linear model of mortality (solid black line; SI) artificially lower the age-specific probability of death q_x (points) in late life, causing late-life mortality deceleration and plateaus (dotted lines). (B) These simulated effects often reflect late-life mortality patterns observed in real data, for example shown here in Jeanne Calment's birth cohort (orange). Introducing age-coding errors, by randomly re-assigning individuals between observed cohorts, (B) further increases rates of mortality deceleration, (C) increases proportionally larger errors in the calculated probability of death, and (D) greatly reduces the probability of death at advanced ages. Errors generated with a probability p = 0.001 (grey) and p = 0.0001 (black), data in (A) fit by locally smoothed splines (dashed lines).

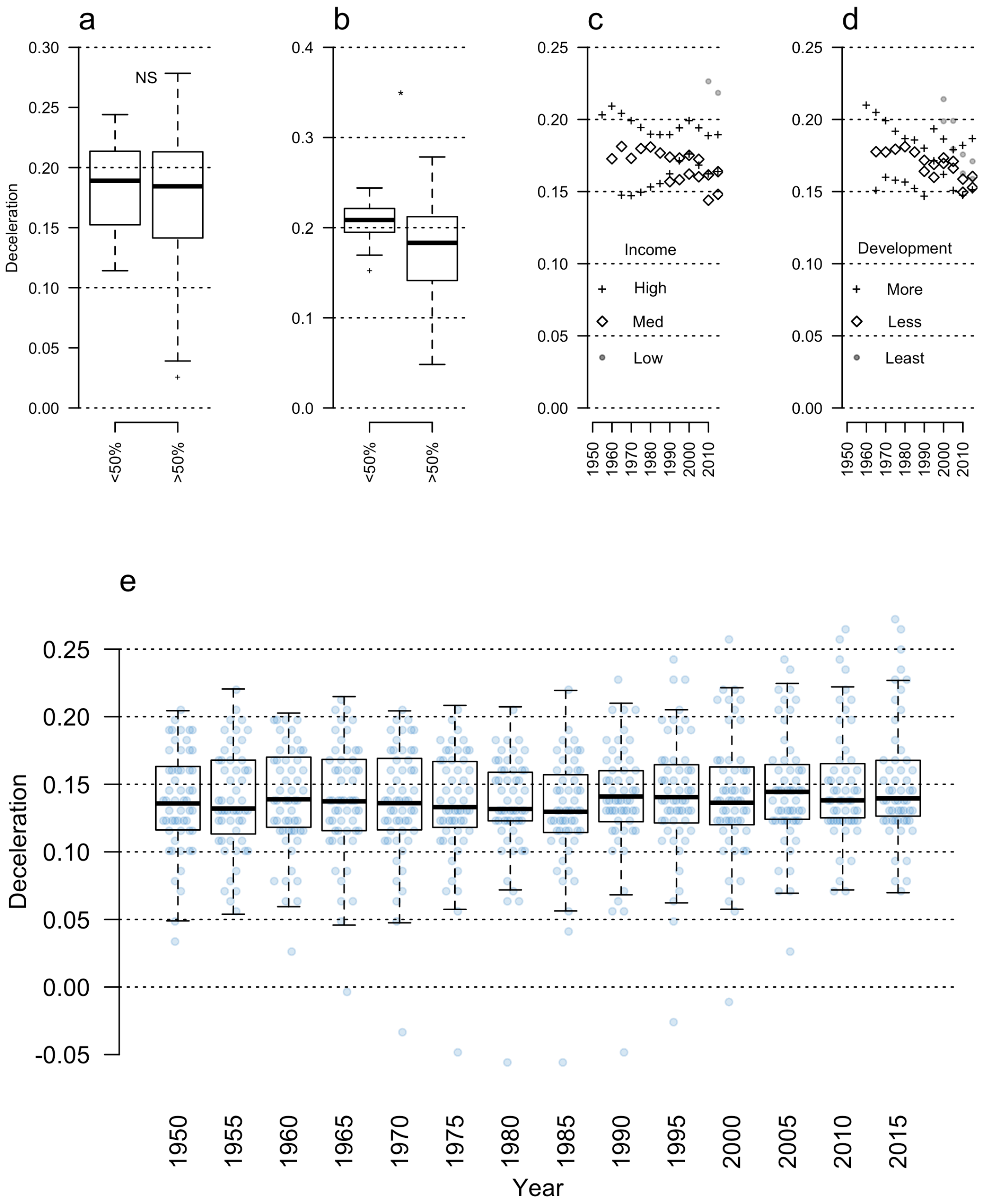

Reduced late-life mortality decelerations in populations with better population data and higher vital statistics coverage. The rate of late-life mortality deceleration (y-axis) is not linked to differences in (A) the fraction of the population with death certificates as in Fig 3A. However, late-life mortality deceleration is still linked to (B) the fraction of the population with birth certificates, (C) per capita gross domestic product and (D) levels of population development (Bonferroni-corrected pairwise t-test; asterisk indicates $p = 0.01$). In populations with continuous records of late-life mortality (E), mortality deceleration rates have not fallen since 1950. However, see lifespan-corrected data on page 4 below that indicate lifespan-adjusted reductions in LLMD.

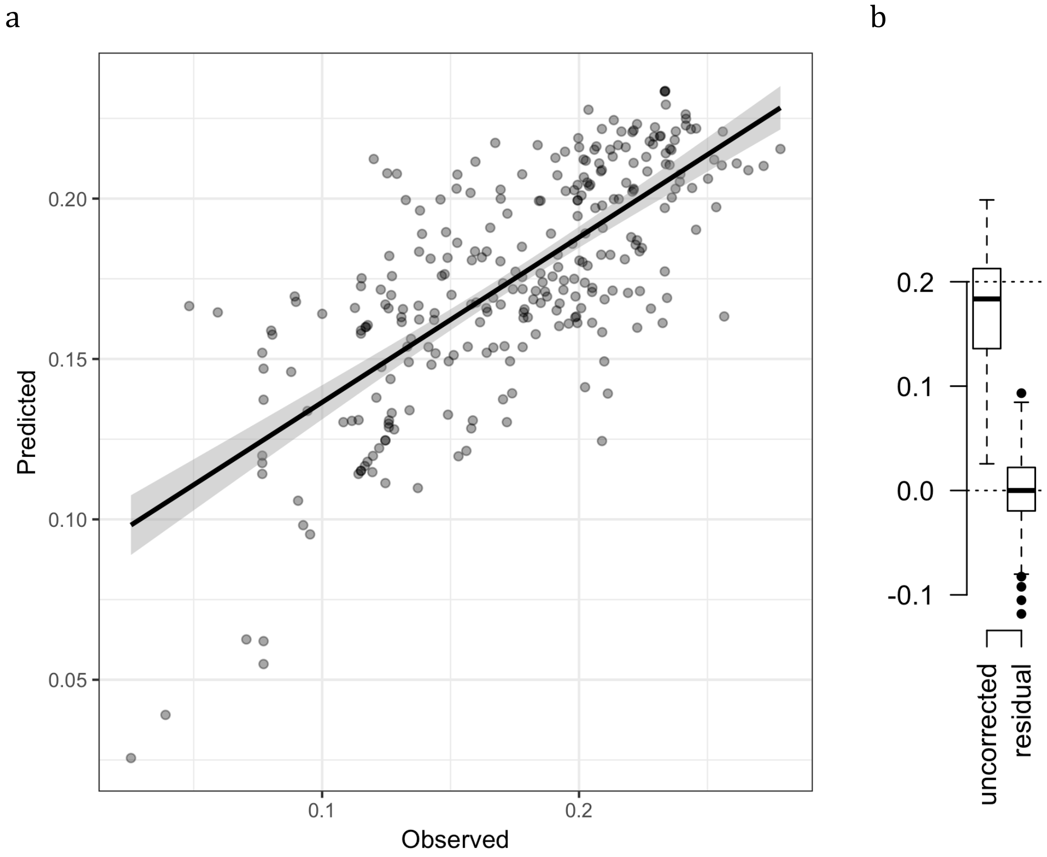

Late-life mortality deceleration predicted by a mixed linear model. A mixed linear mixed model (A) constructed using predictors of sampling error rates and continent of sampling explains the majority of human variation in late-life mortality deceleration (Pearson's r = 0.71; adjusted R^2 =0.36; $p<10^{-6}$; N = 211 populations). (B) Correction for these factors eliminates late-life mortality decelerations in humans.

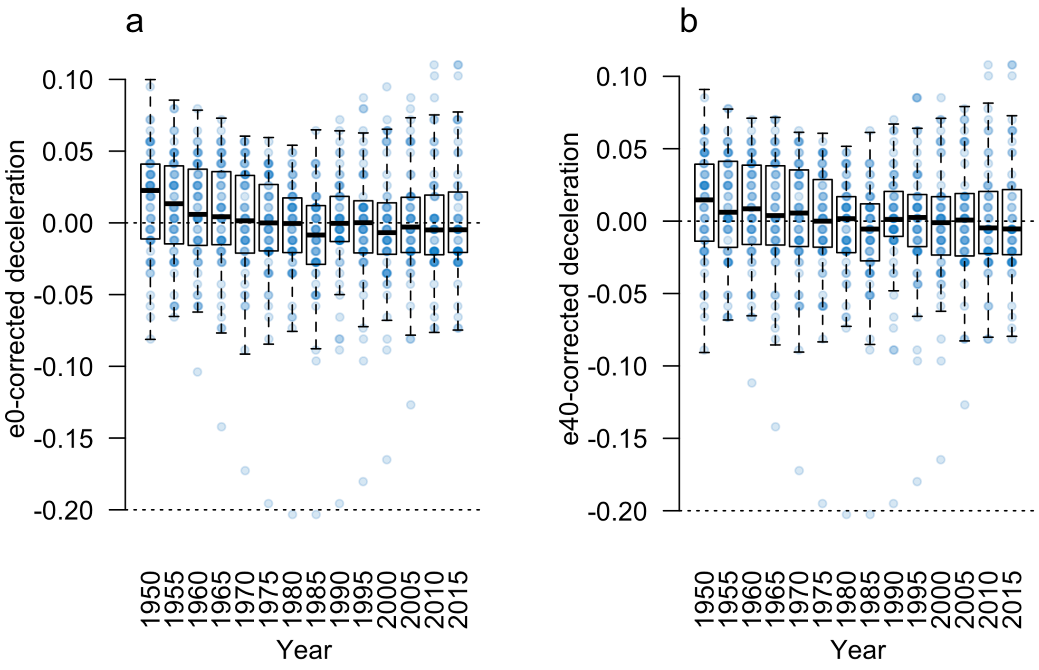

The effect of correcting for average and adult life expectancy variation on late-life mortality decelerations. Correcting for the weak interaction of average life expectancy (A) or adult life expectancy (B) with late-life mortality deceleration has limited effect on the capacity of data quality to predict mortality rate decelerations. However, post-corrected data may indicate a weak historical trend absent in uncorrected hazard rates. Uncorrected data is shown on page 4.
